# Evaluation of Hypolipidaemic Activity of *Capparis decidua*

**Published:** 2009-03

**Authors:** Neelkamal Chahlia

**Affiliations:** *MDPG College, Sriganganagar, Rajasthan, India*

**Keywords:** *Capparis decidua*, diabetes, hypolipidemic

## Abstract

**Background:**

The effect of various extracts (50% ethanolic) of *Capparis decidua* on lipid profile of streptozotocin diabetic rats was studied.

**Procedure:**

The extract was administered to the diabetic models for 30days.

**Findings:**

The extract produced a significant (p<0.05) dose-dependent decrease in the levels of total cholesterol (TC), Triacylglycerol (TG), low-density lipoprotein-cholesterol (LDL cholesterol), with a significant increase in the level High-density lipoprotein-cholesterol (HDL-C).

**Conclusion:**

The extracts of *Capparis decidua* prove to have a hypolipidemic potential.

## INTRODUCTION

The use of medicinal plants in the management of various illnesses is due to their phytochemical constituents and dates back to antiquity (1). However, during the last decade, an increase in the use of medicinal plants has been worldwide (2).

Heart diseases have been implicated as leading causes of death for both men & women of all racial and ethnic groups. Diabetes mellitus is a disease characterized by hyperglycaemia, and hyperlipidaemia which leads to an increased risk of atherosclerosis and other cardiovascular diseases (3). A number of plants have been used traditionally in the treatment of various cardiovascular diseases (4). The mechanisms by which *Capparis decidua* produces antidiabetic effects has been studied but the present study was carried out to evaluate the hypolipidemic effects of different parts of *Capparis decidua* in STZ-induced diabetic rats, who were not fed atherogenic diet.

*Capparis decidua* belongs to family Capparidaceae. It is commonly found in the dry regions in India, Pakistan, Egypt and Tropical Africa. It’s a struggling, glabrous shrub. The bark has an acrid, sharp, hot taste; analgesic, diaphoretic, alexeteric, laxative, anthelminthic; good in cough and asthma, ulcers and boils, vomiting, piles and all inflammations (Ayurveda) The bark, under phytochemical investigations revealed the presence of n-pentacosane, n-tricontanol and β-sitaosterol besides a water-soluble alkaloid, 1-stachydrine (5). Besides these, six new phytoconstituents have been isolated and characterized from the root bark, which are capparisterol, Capparideciduasterol, Capparisditerpenol, in aliphatic hydroxyketone and capparisditerpenyl ester (6).

## MATERIAL AND METHOD

### Collection and Identification of Plant Material

The chosen plant, *Capparis decidua* was identified and selected by the experts of Botany Department, J.N.V. University, Jodhpur. For the present study various parts of the *Capparis decidua* i.e., fruits, flowers and barks were collected in and around Jodhpur (India).

### Preparation of Plant Extract

The collected plant material was shade dried and subjected to Soxhlet extraction with 50% ethyl alcohol. The aqueous-alcohol extract solution is then concentrated under reduced pressure until most of the methanol had evaporated and a brownish crude extract was obtained.

Extract was stored in sterile glass containers at –4°C.

### Experimental Animals

After getting approval from the Institutional Animal Ethical Committee, albino rats, Rattus norvegicus of Sprague Dawley strain, weighing about 150 to 200 gm were selected from our inbred colony and were used for the experiment. They were housed in polypropylene cages measuring 12” × 10” × 8” under controlled temperature conditions (25 ± 2°C) with 12:12 hrs light and dark cycle. Animals were fed on balanced diet of soaked maize, wheat and chiken beans supplemented with multivitamins and water ad libitum.

Animals were regularly checked throughout the investigation for any infection and if found infected, the animals were isolated and treated. A total check of cleanliness of the cages and general environment of animal house was kept. Animals were treated intermittently with antibiotic and antihelminthic suspensions as a prophylactic measure.

### Induction of Diabetes

Diabetes was induced in rats that had been fasted for 24 hours by intraperitoneal injection of streptozotocin (Sigma chemicals Co., St. Louis, MO, U.S.A.) freshly dissolved in citrate buffer (pH4.5) immediately before use. Streptozotocin was given at a dose of 65 mg/Kg body weight (7). The streptozotocin treated animals were given 5% glucose solution for 24 hours following streptozotocin injection to prevent initial drug induced hypoglycaemic mortality (8).

### Experimental design

The experimental models were administered various plant extracts for a period of 30 days. The control and experimental groups consisted of 10 animals each, but mortality was higher in streptozotocin diabetic rats and so mean of 5values was taken. The study consisted of following groups:

- Group 1: Control or Intact: They received drug vehicle only i.e. normal saline water (2 ml/kg body weight/day);
- Group 2: Diabetic control;
- Group 3: Diabetic + *Capparis decidua* bark extract treatment;
- Group 4: Diabetic + *Capparis decidua* flower extract treatment;
- Group 5: Diabetic + *Capparis decidua* fruit extract treatment.

### Acute toxicity studies

The acute toxicity test (LD50) of the extract was determined according to the OCED test guidelines No.420 (Organization for Economic Co-operation and development).

### Drug Administration

The various extracts of *Capparis decidua* were prepared for oral administration by dissolving it in normal saline. The extract was fed at an effective dose of 500 mg/kg body weight.

Twenty four hours after the last administration, the animals were anaesthetized with chloroform vapor and dissected. Whole blood was obtained by cardiac puncture from each rats and collected into sample bottles. The serum and tissue samples (liver, heart and adrenal gland) were kept at -20°C until assayed for Biochemical parameters.

Serum total cholesterol, triglyceride and High density Lipo-protein (HDL) were measured by enzymatic colorimetric method using Randox kits. The concentration of low density lipoprotein (LDL) cholesterol was calculated by the formula of Friedwald *et al*. (9). Frozen tissues were analyzed for quantitative estimation. Cholesterol was estimated in liver, heart muscle and adrenal gland by method of Zaltkis *et al*., 1953 (10).

### Statistical Calculations

All the values were expressed in terms of mean value ± standard error. The different groups were compared among each other using student “t” test (11). The results were analyzed for statistical significance using ANOVA test.

## RESULTS AND DISCUSSION

The aim of the present study was to test the effect of the *Capparis decidua* extracts on serum & tissue cholesterol and triglyceride concentrations. It has been previously reported that *Capparis decidua* exhibited a hypoglycaemic and antioxidant activity in STZ rats (12). Administration of *Capparis decidua* extract to diabetic animals normalizes blood glucose concentration and reduces triglyceride levels

A highly significant increase in serum cholesterol was observed in Group 2 (Table 1).Significant reduction (P≤0.01) were seen in Group 3 and Group 5 as compared to Group 4, having slightly significant reduction in the level of Serum cholesterol. The ethanolic extracts of *C.decidua* produced a slightly significant (p<0.05) decrease in the levels of total cholesterol, triglyceride, LDL-cholesterol and VLDL-cholesterol of the extract treated group compared to diabetic control. There was a slightly significant (p<0.05) increase in the levels of HDL-cholesterol as compared to diabetic control. The levels of LDL cholesterol were reduced significantly (P≤0.01) by the Bark and Fruit extracts, but *Capparis decidua* flower extract showed only slightly significant reduction (P≤0.05) (Table 1).

**Table 1:** The Effects of the different crude extracts of *Capparis decidua* on serum cholesteroland triglyceride levels in fasting normoglycaemic and STZ induced hyperglycaemic rats (Mean of 5 Values ± SEM)

Treatment groups	Cholesterol (mg/dl)	Triglycerides (mg/dl)	HDL (mg/dl)	LDL (mg/dl)	VLDL (mg/dl)

Intact Control (Group1)	89.36 ± 0.432	66.68 ± 0.912	59.6 ± 0.45	78.2 ± 0.19	21.8 ± 0.36
Diabetic Control (Group 2)	201.50^c^ ± 0.58	149.20^c^ ± 0.56	14.9^c^ ± 0.29	144^c^ ± 0.67	47.8^c^ ± 0.30
Diabetic + *C.decidua* bark extract treatment (Group 3)	96.10^a^,^f^ ± 0.89	76.70^a^,^f^ ± 0.35	56.46^a^,^g^ ± 0.22	85.84^a^,^f^ ± 0.22	22.1^d^,^f^ ± 0.32
Diabetic + *C.decidua* flower extract treatment (Group 4)	125.4^b^,^e^ ± 0.67	80.63^a^,^f^ ± 0.42	46.02^b^,^g^ ± 0.30	126.42^b^,^e^ ± 0.72	34.26^a^,^f^ ± 0.39
Diabetic + *C.decidua* fruit extract treatment (Group 5)	98.00^a^,^f^ ± 0.34	76.20^a^,^f^ ± 0.72	44.2^b^,^g^ ± 0.19	92.6^a^,^f^ ± 1.28	22.9^d^,^f^ ± 0.33

Group 2, 3, 4 and 5 were compared with Group1:

a P ≤ 0.05,

b P ≤ 0.01,

c P ≤ 0.001,

d Non-significant;

Group 3, 4 and 5 were compared with Group2:

e P ≤ 0.05,

f P ≤ 0.01,

g P ≤ 0.001.

The results demonstrated that the ethanolic extracts of *Capparis decidua* induced a significant decrease of plasma cholesterol levels in STZ-diabetic rats. Some studies have reported a similar lipidemic-lowering activity of some medicinal plants (13, 14). The reduction was more pronounced in bark and fruit extract.

Induction of diabetes in normal rats resulted in an increase in cholesterol content of liver, heart and adrenal (Table 2). The reduction in the levels of liver cholesterol by the extract of *Capparis decidua* was highly significant (P≤0.001). As reported earlier by Mutalik *et al*., 2005 (15). A significant (P≤0.001) decrease in the level of cholesterol of heart was seen in Flower and Fruit extract treatment groups as compared to bark extract, where a non-significant decrease was observed. The elevated levels of cholesterol in the diabetic control group were significantly lowered in 30 days treatment group of *Capparis decidua* extracts. Similar results were obtained by Ananthan *et al*., 2003 (16).

**Table 2:** Tissue biochemistry of 30 days treatment of various extracts of Capparis decidua in albino rats (type 1 diabetes) (mean of 5 values ± SEM)

Treatment groups	Cholesterol (mg/gm)		
	Liver	Heart	Adrenal

Intact Control (Group1)	13.82 ± 0.37	6.49 ± 0.16	24.06 ± 0.66
Diabetic Control (Group 2)	27.46^b^ ± 0.41	8.6^b^ ± 0.28	31.24^b^ ± 0.76
Diabetic + *C. decidua* bark extract treatment (Group 3)	14.36^c^,^e^ ± 0.22	8.09^a^,^f^ ± 0.46	24.02^c^,^e^ ± 0.39
Diabetic + *C. decidua* flower extract treatment (Group 4)	13.93^c^,^e^ ± 0.09	7.15^c^,^d^ ± 0.18	23.01^c^,^e^ ± 0.29
Diabetic + *C. decidua* fruit extract treatment (Group 5)	13.81^c^,^e^ ± 0.17	7.09^c^,^d^ ± 0.37	24.25^c^,^e^ ± 0.76

Group 2, 3, 4 and 5 were compared with Group1:

a P ≤ 0.01,

b P ≤ 0.001,

c Non-significant;

Group 3, 4 and 5 were compared with Group2:

d P ≤ 0.01,

e P ≤ 0.001,

f Non-significant.

Hyperlipidemia has been implicated in the development of atherosclerosis (17).

The underlying mechanism of the lipidaemic-lowering activity of *Capparis decidua* could be the inhibition of lipid absorption due to the presence of saponins and tanins in the ethanolic extract (18); hence used as hypocholesterolemic. It may operate through increased fecal excretion of cholesterol as well as bile acids (19).

Oral administration of saponins from some medicinal plants, significantly reduce triglycerides and cholesterol levels in rat. The usage of diet with high saponins contents is also suggested to reduce heart diseases (20).

HDL functions in the transport of cholesterol away from the peripheral tissues to the liver, thus preventing the genesis of atherosclerosis. The observed significant increase in the level of HDL, further points to the cardiac protective activity of the extracts.

## CONCLUSION

In the present study, the extracts of *Capparis decidua* prove to have a hypolipidemic potential. Further investigations are warranted to identify the hypolipidemic active principles and elucidate their mechanism of action.

## References

[1] Yakubu MT, Akanji MA, Oladiji AT (2007). Male sexual dysfunction and methods used in assessing medicinal plants with aphrodisiac potentials. Pharmacog. Rev.

[2] Faizal P, Suresh S, Satheesh R, Augusti KT (2009). A study on the hypoglycaemic and hypolipidemic effects of an ayurvedic drug Rajanyamalakadi in diabetic patients. Indian Journal of Clinical Biochemistry.

[3] Ogbonnia SO, Odimegwu JI, Enwuru VN (2008). Evaluation of hypoglycaemic and hypolipidaemic effects of aqueous ethanolic extracts of *Treculia africana* Decne and *Bryophyllum pinnatum* Lam. and their mixture on streptozotocin (STZ)-induced diabetic rats. African Journal of Biotechnology.

[4] Walid SQ (2008). Hypolipidaemic effects of Ballota undulate in Rabbits. Pakistan Journal of Biological Sciences.

[5] Yadav P, Sarkar S, Bhatnagar D (1997). Action of *Capparis decidua* against alloxan-induced oxidative stress and diabetes in rat tissues. Pharmacol Res.

[6] Gupta J, Ali M (1998). Phytoconsitituents of *Capparis decidua* root barks. J. Medicinal Aromatic plant sci.

[7] Theodorou NA, Vrbova H, Tyhurst M, Howell SL (1980). Management of intestinal amoebiasis by an indigenous drug Kantaki Karanja (*Caesalpinia crista* L.). Diabetologia.

[8] Andallu B, Varadacharyulu N (2002). Ch. Control of hyperglycemia and retardation of cataract by mulberry (*Morus indica* L.) leaves in streptozotocin diabetic rats. Ind. J. Exp. Bio.

[9] Friedwald WT, Levy RT, Frederickson DS (1972). Estimation of the concentration of low lipoprotein cholesterol in plasma without use of preparative ultracentrifuge. Clin. Chem.

[10] Zaltkis A, Zak B, Boyle AJ (1953). A new method for the direct determination of serum cholesterol. J. Lab. Clin. Med.

[11] Ipstein J, Poly F (1970). In: Banchroft’s introduction to biostatstics.

[12] Gaind KN, Juneja TR, Jain PC (1969). Anthelmintic and Purgative Activity of *Capparis decidua* Edgew. Indian J. Hosp.Pharm.

[13] Ram A, Lauria P, Gupta R, Kumar P (1997). Hypocholesterolemic effects of Terminalia arjuna tree bark. J. Ethnopharmacol.

[14] Sharma SR, Dwivedi SK, Swarup D (1997). Hypoglycaemic, antihyperglycaemic andhypolipidemic activities of Caesalpinia sylvestreseeds in rats. J. Ethnopharmacol.

[15] Mutalik S, Chetana M, Sulochana B, Devi PU (2005). Effect of Dianex, a herbal formulation on experimentally induced diabetes mellitus. Phytother.

[16] Ananthan R, Latha M, Ramkumar KM, Pari L (2003). Effect of *Gymnema montanum* leaves on serum and tissue lipids in alloxan diabetic rats. Expr. Diabesity Res.

[17] Kaplan NM (1989). The deadly quarter; Upper body weight, glucose intolerance hypertriglyceridemia and hypertension. Acta. Int. Med.

[18] Goyal R, Grewal RB (2003). The influence of Teent (*C.decidua*) on human plasma triglycerides, total lipids and phospholipids. Nutr. Health.

[19] Agarwal V, Chavan BM (1988). Plant Foods Hum. Nutr.

[20] Oakenfull D (1981). Saponins in food. Food Chem.

